# Conditioned pain modulation, cortical imbalance, and arthrogenic muscle inhibition: an integrative model with implications for exercise in chronic pain

**DOI:** 10.3389/fpain.2026.1928053

**Published:** 2026-08-12

**Authors:** Juan Vicente-Mampel, Montserrat Mampel-Ariño, Ciara Vicente-Mampel, Mariola Belda-Antolí, Marta Martinez-Soler, Mónica Alonso-Martín

**Affiliations:** 1 School of Medicine and Health Science, Department of Physiotherapy, Catholic University of Valencia, Torrent, Valencia, Spain; 2 OH Center Sport Castelló, Castellón de la Plana, Castellon, Spain; 3 Assistant Professor (PhD) in Commercial Law, Department of Private Law, Universitat Jaume I de Castelló, Castellón de la Plana, Spain

**Keywords:** arthrogenic muscle inhibition and neuromodulation, chronic pain, conditioned pain modulation, exercise, neuromodulation

## Abstract

**Objective:**

Conditioned Pain Modulation (CPM), cortical excitability, psychosocial factors, and arthrogenic muscle inhibition (AMI, defined as reflex inhibition of musculature surrounding a damaged joint), proposing a framework linking these domains in chronic pain.

**Methods:**

A narrative literature search was conducted in databases, focusing on studies addressing endogenous pain modulation, cortical mechanisms, psychosocial influences, and motor dysfunction in chronic musculoskeletal conditions.

**Results:**

Evidence suggests that impaired CPM is associated with altered cortical function, including compromised prefrontal top-down control and maladaptive motor cortex excitability. In this context, AMI reflects peripheral joint-driven adaptations and centrally mediated alterations in motor output. We propose that CPM and AMI interact through a shared cortical-motor axis, where the dorsolateral prefrontal cortex acts as an executive top-down regulator of brainstem nociceptive pathways, while the primary motor cortex serves as a crucial neurophysiological intersection actively engaged during CPM paradigm, collectively contributing to persistent AMI.

**Discussion:**

Chronic pain involves complex interactions among pain modulation, cortical mechanisms, and motor control. Consequently, baseline CPM efficiency may help predict AMI severity, offering a novel perspective for early patient identification and stratified, timely multimodal rehabilitation; however, further empirical research is required.

## 1 Introduction

Chronic pain imposes substantial physical, psychological, and socioeconomic burdens (1). Its prevalence increases progressively with age and is frequently linked to functional impairment, depression, and reduced quality of life (2). While chronic pain states are heavily influenced by central sensitization (CS)—the hyperexcitability of central nociceptive pathways characterized by enhanced spinal signaling and pain amplification (3, 4)—an individual's clinical presentation is equally determined by the integrity of their endogenous descending pain modulation. Critically, CS operates through neurophysiological mechanisms that are distinct from descending inhibition, meaning no direct causal link exists between baseline pain-modulation capacity and the presence of CS; pain-free individuals can naturally exhibit either a predominantly inhibitory or facilitatory pain profile (5). It is therefore vital to distinguish between clinical pain amplification driven by sustained CS and the transient, facilitated pain responses occasionally observed during psychophysical testing. Because descending pathways typically mitigate nociceptive input, deficiencies in these inhibitory networks are highly prevalent in chronic musculoskeletal conditions (6), highlighting the clinical need to systematically assess and understand these modulatory systems.

Conditioned pain modulation (CPM) serves as a validated framework for evaluating the effectiveness of endogenous pain inhibitory mechanisms (7). Methodologically, CPM is assessed through a psychophysical “pain-inhibits-pain” paradigm, where a painful test stimulus is evaluated before and during the application of a distant, noxious conditioning stimulus (8). It corresponds to the central nervous system's ability to attenuate nociceptive input (9–11). From a neurobiological standpoint, this descending system relies on a sophisticated top-down modulatory network, where executive cortical structures, such as the prefrontal cortex (PFC) (12), specifically the dorsolateral prefrontal cortex (DLPFC), project to subcortical orchestrators, such as the periaqueductal gray (PAG) (13). The PAG subsequently recruits monoaminergic and opioid pathways via the rostral ventromedial medulla (RVM) to gate nociceptive signaling at the spinal dorsal horn (14). Alterations in the function of these pathways frequently coexist with CS, which is characterized by enhanced neuronal excitability, expansion of receptive fields, and amplification of pain signaling (15). The CPM efficiency varies widely among individuals and is influenced by cortical and subcortical connectivity (16), autonomic nervous system function (17), and psychological factors (18). Several interventions aimed at enhancing endogenous pain inhibition have been explored; however, the available findings remain heterogeneous. During exercise treatment, exercise-induced hypoalgesia (EIH) is activated, with its magnitude appearing to depend on factors such as intensity, duration, baseline pain, and individual characteristics (19–24). Importantly, these exercise-induced mechanisms are often evaluated alongside baseline CPM profiles as complementary paradigms to assess endogenous pain inhibition (25). Despite this growing body of research, the exact neurobiological interactions between these mechanisms remain unclear.

From a motor perspective, arthrogenic muscle inhibition (AMI) is a clinically significant phenomenon, particularly after anterior cruciate ligament (ACL) injury or reconstruction (26). AMI is defined as an involuntary, ongoing neuromuscular impairment characterized by diminished voluntary muscle activation surrounding a damaged joint. It is driven by altered joint afferent discharge due to factors like effusion or inflammation, which triggers central neural adaptations that sustain muscle inhibition (27, 28). This condition contributes to strength asymmetries, gait alterations, compensatory movement patterns, and an increased risk of reinjury (27). AMI has also been documented in other clinical conditions, such as knee osteoarthritis (29), chronic ankle instability (30), anterior knee pain (31), and multifidus dysfunction in chronic low back pain (32). Traditionally, recovery strategies include exercise, cryotherapy, and transcutaneous electrical nerve stimulation (TENS) used in multimodal approaches (33). However, persistent deficits underscore the underlying impairments in functional movements and postural control (34).

In addition to its role in sensory modulation, alterations in pain processing may influence motor control, suggesting a potential mechanistic link between CPM and AMI (35). The anatomical rationale for this potential link resides in the shared brain regions, most notably the primary motor cortex (M1). M1 serves as a critical neural intersection, as it is actively engaged in descending nociceptive regulation during CPM paradigms (36), while simultaneously acting as the primary driver of the diminished corticospinal transmission and voluntary muscle activation (37) seen in AMI**.** Understanding these interactions (pain modulation, cortical connectivity, AMI, and psychological constructs) is essential for optimizing patient rehabilitation (38). Because these domains have largely been studied in isolation, this review aims to synthesize the current evidence and propose an integrated conceptual framework within the context of chronic pain. The central premise of this review is that CPM and AMI may share common cortical substrates involving the DLPFC and M1. Consequently, CPM assessment may provide clinically relevant information regarding susceptibility to persistent AMI and rehabilitation outcomes in patients with AMI.

## 2 Methods

To ensure a comprehensive, transparent, and reproducible synthesis of the literature, a narrative search strategy was executed across the Scopus and Web of Science electronic databases from database inception to March 2026. These specific platforms were selected because of their comprehensive indexing of high-impact clinical neuroscience, rehabilitation, and sports medicine literature, which minimizes selection bias across disciplines. The search architecture was structured using relevant free-text keywords, including “Conditioned Pain Modulation”, “CPM”, “arthrogenic muscle inhibition”, “AMI”, “primary motor cortex”, “motor cortex excitability”, “M1”, “prefrontal cortex”, “PFC”, “central sensitization”, and “psychosocial factors”. These search terms were strictly operationalized around these specific neurophysiological and motor pillars to maintain a tight focus on musculoskeletal conditions and prevent conceptual drift into unrelated systemic neurological disorders.

To strictly control this conceptual drift, search terms within each distinct domain were grouped using the Boolean operator “OR”, while the domains themselves were cross-referenced using “AND”. For instance, to capture the psychological intersection without diluting the search into general pain-distress literature, the strategy was explicitly constrained to (“Conditioned Pain Modulation” OR “CPM”) AND (“Arthrogenic Muscle Inhibition” OR “AMI”) AND (“psychosocial factors” OR “kinesiophobia” OR “catastrophizing”). Similarly, the motor-sensory intersection was isolated using the following keywords: (“Conditioned Pain Modulation” OR “CPM”) AND (“Arthrogenic Muscle Inhibition” OR “AMI”) AND (“primary motor cortex” OR “M1” OR “dorsolateral prefrontal cortex” OR “DLPFC” OR “cortical excitability”). The inclusion criteria targeted peer-reviewed experimental studies, randomized controlled trials, cohort studies, and critical reviews published in English that addressed the neurophysiological intersection between endogenous pain modulation and motor system regulation. Document screening and data extraction focused on synthesizing the mechanisms of central pain processing, corticospinal excitability, exercise-induced hypoalgesia, and noninvasive brain stimulation models to inform the proposed integrative framework. Crucially, the formulation, structural design, and reporting quality of this narrative review were strictly aligned with the Scale for the Assessment of Narrative Review Articles (SANRA) checklist to ensure methodological rigor and minimize potential reporting bias (39).

## 3 Neurophysiological basis of CPM

### 3.1 Modulation

Nociception is traditionally categorized into four distinct stages: transduction, transmission, modulation, and perception (40). The modulation stage involves a sophisticated neurochemical equilibrium in the spinal dorsal horn (41). While local nociceptive transmission is facilitated by excitatory mediators such as glutamate and substance P, it is counterregulated by local inhibitory interneurons releasing gamma-aminobutyric acid (GABA) and supraspinal descending monoaminergic projections utilizing serotonin and noradrenaline (42). In this physiological framework, CPM represents the functional and psychophysical expression of these central descending inhibitory networks (43). The inhibitory CPM effect reflects the extent of pain reduction during or after the introduction of a heterotopic noxious conditioning stimulus (9, 44). As noted previously, this functional architecture is driven by an executive network involving the PFC, DLPFC, PAG, and RVM (45–47). Within this network, downstream monoaminergic and endogenous opioid signaling blunt the excitability of wide-dynamic-range spinal neurones to suppress central nociceptive transmission (48). Furthermore, CPM must be conceptualized as a multidimensional, integrative process tightly coupled with cognitive, emotional, and sensory discriminative domains (49). In chronic pain states, CPM efficiency is frequently compromised, reflecting an endogenous shift toward facilitatory pain profiles or disrupted inhibitory capacity that facilitates pain chronification and persistent CS (3, 9). Emerging evidence underscores that this failure in descending pain modulation is intimately intertwined with altered motor cortical excitability (50). Neuromodulatory paradigms targeting the motor cortex have revealed widespread systemic effects on pain thresholds (14), suggesting that distributed sensorimotor networks actively interface with pain regulation circuitries (51). Consequently, sensorimotor integration may be conceptualized as a potential functional interface linking motor processing and pain regulation in chronic pain (52). This interface relies on central systems that simultaneously regulate motor control and pain processing (53). Clinically, this implies that an inefficient baseline CPM could serve as a candidate biomarker to predict severe AMI development, offering a window for timely and individualized rehabilitation efforts. Importantly, the mechanistic link between baseline CPM impairment and AMI onset or severity remains largely inferential. To date, no empirical studies have directly quantified this association, and the proposed link relies on indirect neurophysiological and clinical readouts. While this limits immediate causal assertions, it provides a highly fertile ground for future prospective and experimental verifications.

### 3.2 Cortical mechanisms: prefrontal and motor cortex

Supraspinal pain regulation is heavily governed by precise cortical mechanisms, predominantly concentrated within the PFC and M1 (54). Executive structures, such as the DLPFC, are essential for supervising top-down inhibition (55) and engaging in processes related to selective attention, cognitive expectancy, and affective valuation of nociceptive input (54). For instance, positive cognitive expectancy and placebo conditions can markedly boost CPM outcomes, a phenomenon that is directly mirrored by heightened prefrontal metabolic activity (56). Consequently, distinct prefrontal neuroplastic adaptations or dysfunctions may jeopardize descending inhibition in chronic musculoskeletal disorders, potentially contributing **to** central hypersensitivity. M1 is intrinsically linked to pain regulation and functionally intersects with the governance of corticospinal excitability and motor drive (50). This dual role makes the M1 the primary hub bridging both domains: while its activation may enhance CPM (57), its diminished corticospinal transmission may drive the voluntary activation deficits seen in AMI (31, 53). Conversely, in clinical populations with persistent pain, notable disruptions in motor cortex excitability, characterised by the uncoupling of facilitatory and intracortical inhibitory networks within fronto-motor loops, have been robustly described (58). This aberrant excitability in chronic states supports the notion that maladaptive motor circuit plasticity underpins pain-related motor behaviours and clinical muscle inhibition. This is further substantiated by resting-state functional connectivity data in conditions such as fibromyalgia (FM), where uncoupled M1–PFC communication directly correlates with severe clinical impairments in the endogenous descending pain modulatory system (59, 60).

## 4 AMI and CPM

Functionally, AMI is characterized by an acute, involuntary reduction in muscle activation surrounding an injured or inflamed joint to protect its structure (61). In contrast, chronic AMI impairment occurs when this protective reflex persists after the initial joint pathology resolves, leading to long-term neuromuscular deficits (27). Traditionally, AMI impairment has been interpreted from a narrow peripheral perspective, wherein joint injury, swelling, or inflammation diminishes voluntary muscle activation. However, contemporary neurophysiological evidence indicates that AMI likely reflects a combination of peripheral and central mechanisms (61). In this context, the central contributions to AMI include alterations reflecting impaired corticospinal transmission from motor cortical output to muscle force production (31, 53). In addition, AMI involves cortical motor areas and broader descending modulatory systems implicated in both motor control and pain regulation (53). Specifically, these shared systems involve a fronto-motor circuitry, where the prefrontal executive drive regulates descending nociceptive pathways, while the primary motor cortex simultaneously modulates motor output and pain thresholds.

Ultimately, AMI results from intricate interactions between sensory, motor, and central nervous system mechanisms, reflecting multidimensional impairment of neuromuscular control (61). This shared link suggests that individuals with inefficient baseline CPM (poorer pain inhibition) may exhibit a centrally mediated vulnerability that correlates with more severe and persistent AMI impairment. Clinically, the use of CPM efficiency as a non-invasive nociceptive signaling could allow for the early identification of patients at risk of severe AMI. By establishing this neurophysiological intersection, our model bridges the gap between sensory modulation and motor dysfunction, providing a clear rationale for a crucial window for timely, individualized, and multimodal rehabilitation before deep-seated motor deficits occur. Thus, interventions targeting neuromuscular control and motor system function should be considered primary components of treatment (27, 53). Consequently, therapeutic strategies aimed at restoring nociception regulation via pathways evaluated through CPM and normalizing cortical excitability may be critical for optimizing functional recovery (62).

## 5 Psychological and social influences

Psychosocial factors can significantly influence PFC activity and connectivity, thereby modulating the top-down control of pain processing (63). They have also been implicated in the driving mechanisms involved in neuroplastic changes within the central nervous system (64). Specifically, these factors may influence endogenous pain modulation, potentially leading to reduced CPM efficiency and increased vulnerability to CS (65, 66). In individuals with low back pain, pain modulation processes have been directly linked to psychological constructs such as fear-avoidance beliefs and behavioural adaptations (67). Crucially, these cognitive and emotional factors not only impair inhibitory networks but also actively modulate clinical responses to mechanical interventions. For instance, recent randomized controlled trial evidence in patients with FM demonstrated that baseline pain catastrophizing acts as a key moderator of treatment efficacy, where high levels of catastrophizing significantly diminished the short-term hypoalgesic effects of dry needling on pressure pain thresholds (68).

Beyond sensory modulation, psychosocial constructs heavily influence motor behaviour and neuromuscular function (69, 70). This is highly evident in injured competitive athletes, where cluster analyses have revealed that elevated kinesiophobia directly correlates with prolonged recovery times and severely compromised self-perceived readiness to return to sport (71). Mechanistically, psychological distress alters fronto-motor loops, affecting corticospinal excitability and voluntary muscle activation, thereby contributing to motor control deficits (72). Therefore, psychosocial factors may modulate the interactions between descending pain modulation, cortical excitability, and motor output (73). Notably, direct empirical research exploring how psychological factors modulate AMI remains virtually nonexistent. However, given that kinesiophobia and catastrophizing impair both prefrontal top-down CPM networks and M1 motor drive (74), investigating the direct impact of these psychological constructs on AMI severity represents a critical avenue for future studies**.** Consequently, improving clinical outcomes may require integrative approaches that include the simultaneous management of cognitive, emotional, sensory, and motor components (67).

## 6 Exercise-induced hypoalgesia and CPM

Exercise prescriptions using contemporary pain science and biopsychosocial approaches should be emphasized in clinical practice (75). Physical activity is considered an effective intervention for pain modulation, primarily mediated by the phenomenon of EIH (76). This response is characterized by a temporary decrease in pain sensitivity and perception following physical activity (77). Crucially, the primary purpose of examining EIH within this conceptual framework is to strengthen our understanding of the shared cortical-motor pathways with CPM, demonstrating how a prolonged exercise intervention rather than a transient experimental test paradigm may drive sustained neuroplastic adaptations. Specifically, regarding the activation of supraspinal endogenous opioid and monoaminergic descending inhibitory networks (77, 78). Supporting this overlapping mechanism, evidence shows that combining experimental CPM and EIH paradigms does not induce a stronger cumulative inhibitory effect than either alone (19), suggesting that these mechanisms share a common neurobiological substrate. Through these shared centralized mechanisms, EIH-driven clinical benefits act via neurosensory modulation rather than isolated physical changes within the peripheral musculature. Despite its clinical utility, the magnitude and reliability of EIH vary substantially between individuals and populations (79). This overlap remains incomplete and inconsistent, as the magnitude of pain inhibition varies across paradigms and combining exercise with conditioned pain modulation does not necessarily yield cumulative effects (19), suggesting that EIH may involve a combination of distinct central and peripheral contributions, which are further influenced by heterogeneous assessment protocols (80).

In the context of chronic pain management, the hypoalgesic response is highly variable (81). Indeed, EIH may be diminished or reversed by exercise-induced hyperalgesia. This fluctuation is likely influenced by multiple interacting factors, including CS, psychological state, and baseline physical activity level (67). For instance, distinct response phenotypes to exercise have been identified in patients with low back pain (67). This longitudinal heterogeneity is further evidenced by clinical trials showing that while standardized exercise regimens consistently reduce pain intensity over a 12-week period, the temporal trajectories of functional recovery and changes in pain sensitization are highly sensitive to combining active exercise with complementary passive modalities or pain neuroscience education (82). Similarly, in conditions such as FM, EIH may be reduced and may not improve following prolonged exercise programs, despite observed changes in the somatosensory and cingulo-insular networks (6). The inconsistency of EIH and its relationship with CPM suggests that exercise-induced analgesic responses differ significantly between individuals, reflecting heterogeneity in endogenous pain modulatory capacity. Evidence indicates that baseline CPM efficiency may be associated with the magnitude of EIH in some populations (78), while exercise parameters such as intensity and duration further influence hypoalgesic outcomes (83). Nonetheless, exercise should not be avoided because of the fear of acute pain. Instead, targeted exercise must be strategically leveraged as a primary intervention to stimulate these shared descending pathways, simultaneously reducing pain hypersensitivity and restoring motor cortex excitability to recover voluntary activation in patients with persistent AMI. Ultimately, this framework suggests that baseline CPM efficiency may serve as a valuable clinical indicator for predicting individual responses to these prolonged exercise interventions, offering a clear avenue for stratified, neurobiologically targeted rehabilitation.

## 7 Neuromodulation techniques

Beyond exercise therapy, neuromodulation is an additional potential intervention within this framework. Transcranial direct current stimulation (tDCS) and transcranial magnetic stimulation (TMS) are valuable modalities for modulating cortical excitability and pain processing (84). These interventions primarily target M1 and the PFC, which are critical regions involved in descending pain modulation and motor control (31, 60). Stimulation of M1 has been shown to induce analgesic effects, potentially through the modulation of sensorimotor circuits and descending inhibitory pathways, contributing to the regulation of nociceptive transmission (85). This network-level modulation, involving motor and sensory functions, may underlie motor cortex-related analgesia and provide a neurocircuit-based explanation for the effects observed (62).

Regarding specific modalities, tDCS can alter cortical excitability in a polarity-dependent manner, and its application over M1 has shown potential to enhance CPM (57), although some variability in outcomes persists across inconsistent physiological mechanisms (86). Crucially, multi-session active tDCS protocols over M1 have consistently demonstrated clinical efficacy in reducing pain intensity (87), operating primarily via cumulative neuroplastic and motor pathway reorganization over longer intervals (88). Concurrently, TMS, particularly when targeting M1, has demonstrated more consistent effects in reducing clinical pain owing to the functional reorganization of distributed networks involved in sensory, emotional, and motor pain processing (60). Stimulation of the PFC appears to modulate pain perception, with limited and inconsistent effects on the CPM (54). Neuromodulatory techniques applied to the DLPFC have been shown to reduce pain perception primarily by modulating cognitive expectations (89).

From a motor perspective, these techniques may contribute to AMI recovery by modulating corticospinal excitability and improving voluntary muscle activation (31, 53). This mechanical and neurophysiological restoration has been robustly consolidated by recent meta-analytic evidence evaluating transcranial neuromodulation in ACL injury cohorts (90). Specifically, multi-session protocols applying tDCS over the M1 contralateral to the injury, integrated with physical rehabilitation, induce significant adaptations in corticospinal excitability, quadriceps strength, and balance control (90). This high-level evidence supports the notion that targeted neuromodulation can effectively reverse central nervous system impairments and overcome persistent arthrogenic muscle deficits. However, the effectiveness of these interventions depends heavily on individual factors, such as the baseline CPM state, cortical excitability, and presence of CS (91).

## 8 Integrated model

We propose an integrative conceptual framework, the CPM–cortical–motor model, which aims to integrate current evidence linking cortical mechanisms, descending pain modulation, and motor function. In this context, alterations in PFC function may be associated with reduced CPM efficiency, potentially contributing to increased nociceptive input and features of CS (44). However, the directionality and causality of these relationships have yet to be empirically established. Concurrently, alterations in M1 excitability observed in individuals with chronic pain may reflect changes in corticospinal output and motor function (92), including phenomena such as AMI, although the underlying causal mechanisms have not yet been fully elucidated. Psychosocial factors interact with this system by influencing prefrontal activity and top-down regulatory processes.

Consistent with our primary clinical hypothesis, this unified framework positions baseline CPM efficiency as a potential biomarker capable of predicting the severity and persistence of chronic AMI. In interpreting this framework, it is important to note that even among healthy, pain-free adults, baseline endogenous pain modulation varies substantially, with individuals exhibiting distinct inhibitory or even net facilitatory CPM responses. Mechanistically, a disruption in prefrontal descending pain regulation may facilitate a state of central sensitization, potentially limiting M1 motor output and contributing to muscle activation déficits.

**Figure 1:**
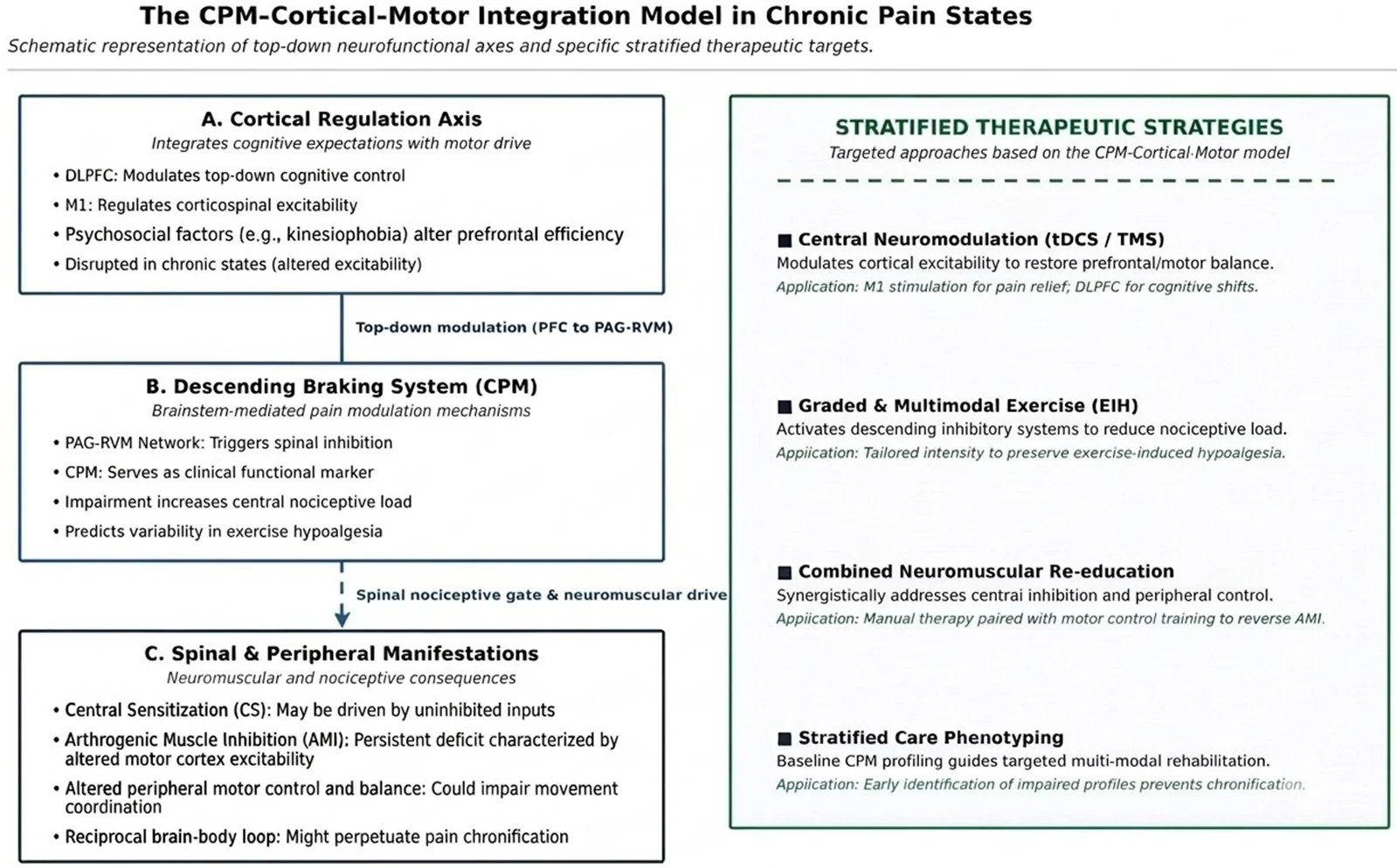
The flowchart illustrates the neurophysiological and clinical interactions between the sensory and motor domains in chronic pain. The continuous line represents the direct descending neurobiological pathway of top-down modulation from the cortical regulation axis to the descending braking system. The dashed line indicates the subsequent downstream path leading to spinal and peripheral manifestations, where impaired endogenous inhibition and central sensitization drive maladaptive neuromuscular outcomes like arthrogenic muscle inhibition. Psychosocial factors (e.g., kinesiophobia) within the cortical regulation axis further influence these central mechanisms and stratified care options.

This model should be interpreted cautiously as a hypothesis-generating framework rather than a definitive mechanistic explanation, representing a simplified conceptualization of complex nonlinear interactions. However, this may support the development of individualized and multimodal interventions for patients with chronic pain. Further research is required to clarify the directionality, underlying mechanisms, and clinical relevance of these interactions, particularly concerning treatment response and patient stratification (Figure 1).

## 9 Clinical implications

Crucially, in alignment with the central theme of this framework, identifying an inefficient baseline CPM allows clinicians to use it as an early predictive biomarker of severe or persistent AMI. Recognizing these high-risk neurophysiological profiles in the acute phase provides an invaluable window for timely, targeted rehabilitation before central motor deficits become established. In this context, CPM serves as a functional marker of descending pain modulation, providing critical insights into the patient's neurophysiological profile and facilitating the identification of distinct pain modulation phenotypes. Accordingly, incorporating CPM assessment into clinical physiotherapy evaluations supports individualized treatment planning within a stratified model of care, where interventions are tailored to the patient's endogenous modulatory capacity and underlying neurophysiological characteristics.

For example, individuals with impaired CPM may exhibit a reduced or variable hypoalgesic response to exercise, suggesting the need for a graded and multimodal approach with careful consideration of exercise intensity, progression, and tolerance. This variability may reflect an underlying dysfunction within the proposed CPM–cortical–motor model, highlighting the clinical importance of targeting the central mechanisms. In this regard, central neuromodulatory interventions can modulate cortical excitability and influence descending inhibitory pathways, potentially enhancing responsiveness to rehabilitation, although robust clinical evidence is limited. Ultimately, integrating multimodal strategies, such as exercise, manual therapy, and non-invasive brain stimulation, may synergistically improve voluntary muscle activation, reverse central neuromuscular inhibition, and optimize functional outcomes by addressing both central pain processing and peripheral neuromuscular control.

## 10 Limitations

However, the available evidence has several important limitations that warrant consideration. First, substantial methodological heterogeneity exists in the assessment of pain modulation, particularly regarding CPM and EIH protocols, which limits the comparability of studies and generalization of findings (93, 94). In addition, the lack of standardization in stimuli, outcome measures, and experimental procedures reduces the consistency and reproducibility of the results (93, 95). Crucially, these methodological discrepancies make it difficult to establish universal clinical cut-offs, thereby directly affecting the reliability of baseline CPM in predicting AMI vulnerability.

Moreover, inherent inter-individual variability—even among healthy, pain-free populations—along with the frequent use of small or highly specific samples, limits the immediate clinical applicability of the current findings, underscoring the need for larger, more robust, and standardized studies. This profound biological variability means that individual modulatory profiles can range from robust inhibition to net facilitation, introducing noise that challenges the predictive accuracy of the proposed model. Finally, the specific mechanistic link between baseline CPM impairment and chronic AMI onset or severity remains largely inferential, as no empirical study has directly and concurrently quantified this association to date. Consequently, the proposed integrative framework is inherently based on indirect neurophysiological evidence and requires direct experimental validation, specifically through prospective longitudinal designs and paired sensory-motor neuromodulation protocols to establish definitive causal relationships and confirm the validity of this candidate biomarker framework.

## 11 Future directions

Future research should aim to elucidate the direct mechanistic relationship between CPM and AMI through well-designed longitudinal and interventional studies. Specifically, prospective studies integrating neurophysiological, motor, and psychosocial assessments are needed to validate and refine the proposed CPM–cortical–motor model. To achieve this, future trials should concurrently quantify baseline CPM efficiency and acute-to-chronic AMI progression in musculoskeletal cohorts, specifically mapping the corticospinal excitability of the M1 -corticospinal axis using paired-pulse transcranial magnetic stimulation. Additionally, these studies must rigorously control for psychological confounding variables, such as kinesiophobia and pain catastrophizing, to isolate pure neurobiological interactions between descending nociceptive pathways and motor transmission deficits. Furthermore, the integration of CPM assessment into clinical practice warrants further investigation for effective translation, offering a valuable tool for patient stratification and the development of personalized, timely rehabilitation strategies for chronic pain management.

## 12 Conclusions

In conclusion, CPM serves as a candidate biomarker for pain regulation, reflecting the efficiency of descending inhibitory pathways. Cortical mechanisms, particularly within the PFC and M1, along with psychosocial factors such as kinesiophobia and pain catastrophizing, synergistically influence pain perception and motor control. Within this framework, AMI can be conceptualized as a manifestation of integrated peripheral and central dysfunction associated with impaired CPM, altered cortical excitability, and increased nociceptive input. While definitive causal relationships remain to be empirically established, utilizing baseline CPM efficiency as an early predictive biomarker for AMI severity within this integrative model provides a valuable hypothesis-generating framework to guide future research and support the development of individualized, stratified, and **timely** multimodal neurorehabilitation strategies for chronic pain management.
